# Changing trends in clinical research literature on PubMed database from 1991 to 2020

**DOI:** 10.1186/s40001-022-00717-9

**Published:** 2022-06-20

**Authors:** Xiyi Zhao, Hao Jiang, Jianyun Yin, Hongchao Liu, Ruifang Zhu, Shencong Mei, Chang-tai Zhu

**Affiliations:** 1 Department of Transfusion Medicine, Shanghai Jiao Tong University Affiliated Sixth People’s Hospital, No. 600 Yishan Road, Shanghai, 200233 China; 2 Department of Thyroid Breast Surgery, Kunshan Hospital Affiliated to Nanjing University of Traditional Chinese Medicine, Kunshan, 215300 Jiangsu China; 3 Department of Outpatients, Shanghai Jiao Tong University Affiliated Sixth People’s Hospital, Shanghai, 200233 China; 4 Editorial Department, First Hospital of Shanxi Medical University, No 85 Jiefang South Road, Taiyuan, Shanxi China

**Keywords:** Data analysis, Clinical research, Literature summary, PubMed database, Longitudinal analysis

## Abstract

**Background:**

Clinical research publications have become the dominant source and basis of clinical evidence-based decision-making. Exploring the type and quantity of clinical research publications in the PubMed database is useful for clarifying the changing trends of clinical research development in recent years. Therefore, a longitudinal analysis of the type and quantity of clinical research publications in the PubMed database over three decades was conducted.

**Methods:**

The PubMed database was searched to retrieve clinical research according to the type and year of publication from January 1, 1991 to December 31, 2020. The research types were classified as primary and secondary literature.

**Results:**

A total of 1,078,404 primary literatures were retrieved and the constituent proportions were ranked from high to low as case report/series (27.54%), randomized clinical trials (RCTs) (23.62%), cohort studies (21.05%), cross-sectional studies (17.49%), case control studies (9.15%), non-RCTs (1.01%), and pragmatic clinical trials (PCTs) (0.15%). Correspondingly, 1,302,173 secondary literatures were retrieved and ranked as narrative review (70.88%), systematic review (15.02%), systematic review and meta-analyses (13.89%), traditional meta-analyses (4.48%), expert consensus (2.31%), guidelines (1.49%), scoping reviews (0.68%), net meta-analyses (0.40%), and umbrella reviews (0.04%). The average annual growth rate for the primary literature was 10.28%, and ranked from high to low as PCTs (83.68%), cohort studies (17.74%), cross-sectional studies (17.61%), non-RCTs (12.11%), case control studies (8.86%), RCTs (7.68%), case report/series (7.51%); while that for the secondary literature was 10.57%, and ranked from high to low as net meta-analyses (48.97%), umbrella reviews (47.09%), scoping reviews (41.92%), systematic reviews and meta-analyses (33.44%), systematic reviews (33.05%), traditional meta-analyses (12.49%), expert consensuses (9.22%), narrative review (8.72%), and guidelines (2.82%).

**Conclusion:**

Both the composition and number of clinical studies changed significantly from 1991 to 2020. Based on the trend, the case report/series, case control study, and narrative review are on the decline, while cohort study, cross-sectional study, systematic reviews, and systematic review and meta-analysis literature have increased. To improve the quality of clinical evidence, we recommend RCT and cohort study give priority to access to allocated research resources in future.

**Supplementary Information:**

The online version contains supplementary material available at 10.1186/s40001-022-00717-9.

## Introduction

The PubMed database is a free biomedical database developed by the National Center for Biotechnology Information (NCBI), a division of the National Library of Medicine (NLM) and has become a major source of literature for biomedical researchers worldwide due to its convenience, accessibility, and extensiveness [1, 2]. The PubMed database makes it convenient and efficient for researchers and clinicians to study current guidelines, learn about frontier advancements, and identify future research directions [3].

Sacket et al. [4] defined evidence-based medicine (EBM) as “the conscientious, explicit and judicious use of current best evidence in making decisions about the care of individual patients”, and it has become widely accepted due to its strong support of clinical practice guidelines [5, 6]. EBM combines the type of study design with the level of evidence to evaluate the reference value of literatures for clinical practice and scientific research [7, 8]. According to the Grading of Recommendations, Assessment, Development and Evaluations (GRADE) proposed by World Health Organization [9–11], and the Joanna Briggs Institute levels of Evidence and Grades proposed [12], clinical research can be divided into primary and secondary literature. Among the former, randomized clinical trial (RCT) has the highest evidence level, case report/series have the highest risk of bias, and other methods such as cohort or case control studies have intermediate evidence level. Among secondary literature, meta-analyses and systematic reviews that included RCTs have the highest evidence level, expert consensus has the highest risk of bias, and other types of reviews or meta-analyses that included observational study have an intermediate evidence level [13, 14].

A rational allocation of research resources should be conducted to produce more efficient and high-quality results for the advancement of human health; however, the vast amount of research literatures and the multiplicity of study designs pose a great challenge. A longitudinal analysis of the type and quantity of clinical publications can quantitatively measure the constituent proportion and changing trends of various types of studies, and provide reference for the rational allocation of clinical research resources. Although there have been some studies on the changing trends in the literature for specific diseases [15–17], a characterization of the clinical research literature in the field of overall medical health is still lacking. Therefore, our research quantitatively measures the constituent proportion and changing trends of clinical research literatures over three decades.

## Materials and methods

### Literature search and data extraction

**Fig. 1:**
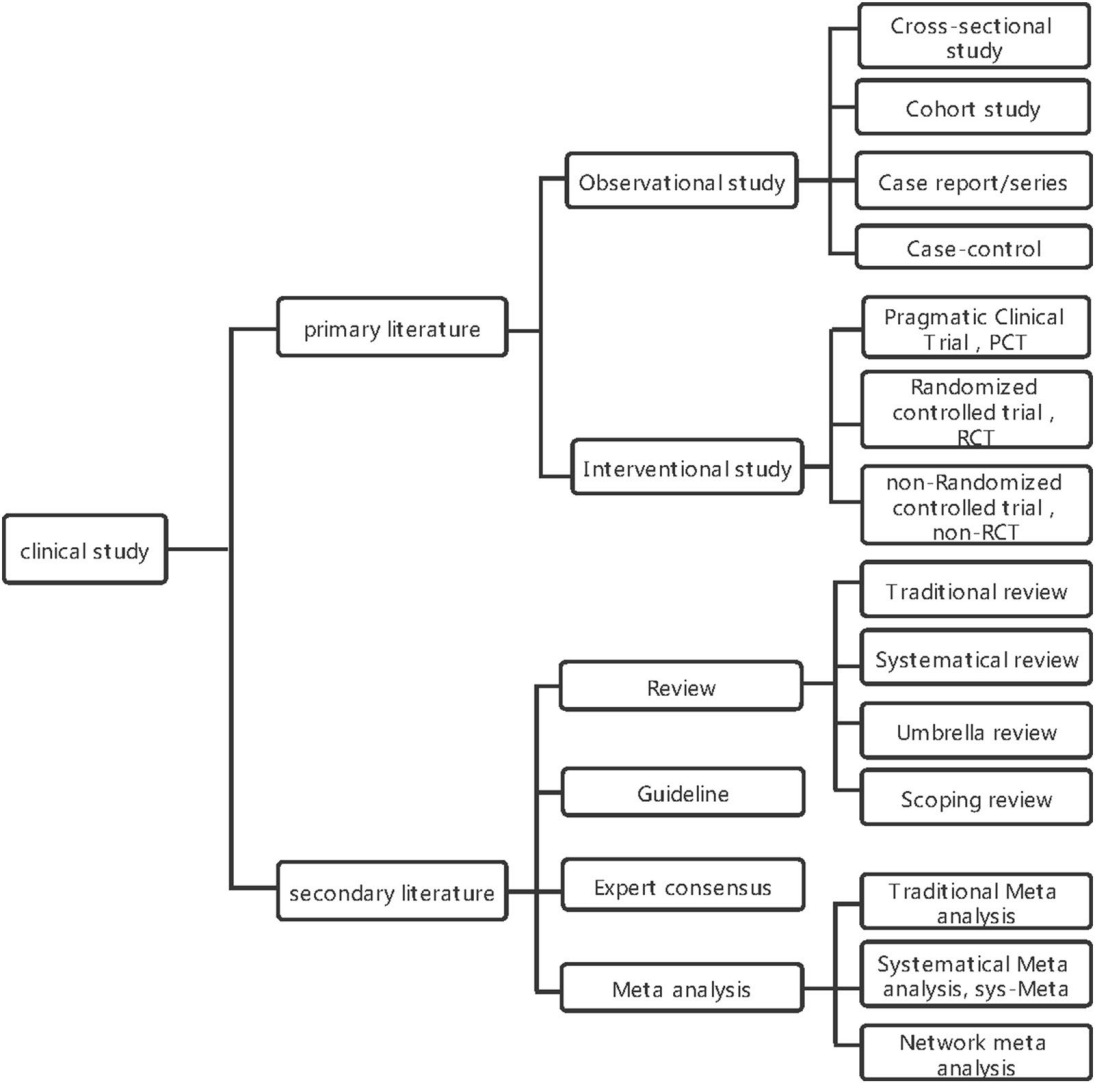
Classification of study type

First, the research type was clarified as primary and secondary literature. In this study, primary literature is defined as observational and interventional studies, with the former consisting of four parts: cross-sectional study, cohort study, case report/series, and case control study, while interventional study consists of RCT, non-RCT, and pragmatic clinical trial (PCT); secondary literature is defined as a type of study that relies on primary literature for further analysis, including guidelines, expert consensus, reviews (narrative, systematic, umbrella, and scoping), and meta-analyses (traditional meta-analysis, systematic review and meta-analysis, and network meta-analysis) [18–20]. The study type classification is presented in Fig. 1. Second, a literature search was conducted using keywords of study type and the filter tool provided by PubMed, which classifies literature according to type, with year of publication from January 1, 1991 to December 31, 2020. By typing “clinical research [all]”, we searched all PubMed literature on clinical research for the last three decades, and by selecting the categories of RCT, PCT, and meta-analysis in the filter tool, combined with the keywords of study type such as network meta-analysis and umbrella review, a specific literature number of various study types during the period 1991–2020 was obtained.

### Statistical analyses

The statistical analyses focused on identifying the changes in quantity, annual growth rate, and 30-year average growth rate of each specific study type in primary and secondary literature between 1991 and 2020; changes in the constituent proportion of each specific study type in primary and secondary literature; and changes in the ratio of the quantity of guidelines or meta-analysis to primary literature.

In this constituent proportion change, we further defined meta-analysis to systematic review and meta-analysis, traditional meta-analysis, and network meta-analysis, while the primary research literature included RCT, PCT, non-RCT, cohort study, and case control study. The annual growth rate = (number of current year publications−number of previous year publications)/number of previous year publications*100%.

The average annual growth rate was calculated using the POWER function included in Microsoft Excel 2019; the specific formula is: the average annual growth rate = POWER [number of publications in year a/number of publications in year b, 1/(a-b)]-1. In this formula, a is the year 2020 and b is the year 1991, and if the number of publications was 0 in 1991 for this study type, then b is the year when the study type first appeared.

The Cochran–Armitage test for trend was used to determine whether the change trend of proportional data was statistically significant [21, 22]. Statistical analyses were performed using GraphPad Prism 6.02 (GraphPad Software, San Diego, CA, USA) and Microsoft Excel 2019.

## Results

A total of 1,078,404 primary literatures were retrieved between 1991 and 2020 and the quantity and constituent proportion were ranked from high to low as 297,045 for case report/series (27.54%), 254,698 for RCT (23.62%), 226,954 for cohort study (21.05%), 188,563 for cross-sectional study (17.49%), 98,653 for case control study (9.15%), 10,871 for non-RCT (1.01%) and 1,620 for PCT (0.15%) (Additional file 1: Table S1). Correspondingly, 1,302,173 secondary literatures were retrieved and ranked as 922,973 for narrative review (70.88%), 195,541 for systematic review (15.02%), 61,246 for systematic review and meta-analysis (13.89%), 58,283 for traditional meta-analysis (4.48%), 30,084 for expert consensus (2.31%), 19,459 for guidelines (1.49%), 8,806 for scoping review (0.68%), 5,216 for network meta-analysis (0.40%), 565 for umbrella review (0.04%) (Additional file 1: Table S2).

Specifically, as for the quantity of publications of primary study, in the year 1991, the total number of case report/series was the highest at 3,320; RCT was second at 2,037; the number of cross-sectional study and cohort study was relatively small at no more than 200. PCT appeared for the first time in 2011, while in the year of 2013, the number of RCTs reached 14,661, for the first time surpassing the 14,551 of case report/series, which was the highest number of primary study in that year. By 2016, the cohort study number had reached 18,004, exceeding RCTs (17,140) for the first time and consistently occupying the top of the primary study list in the following years. Cross-sectional study, being one of the primary study types with the lowest number of publications in 1991, surpassed case control study for the first time in 2005 and then exceeded RCT in 2018, eventually emerging with case report/series as the most primary study type after cohort study in 2020.

**Fig. 2:**
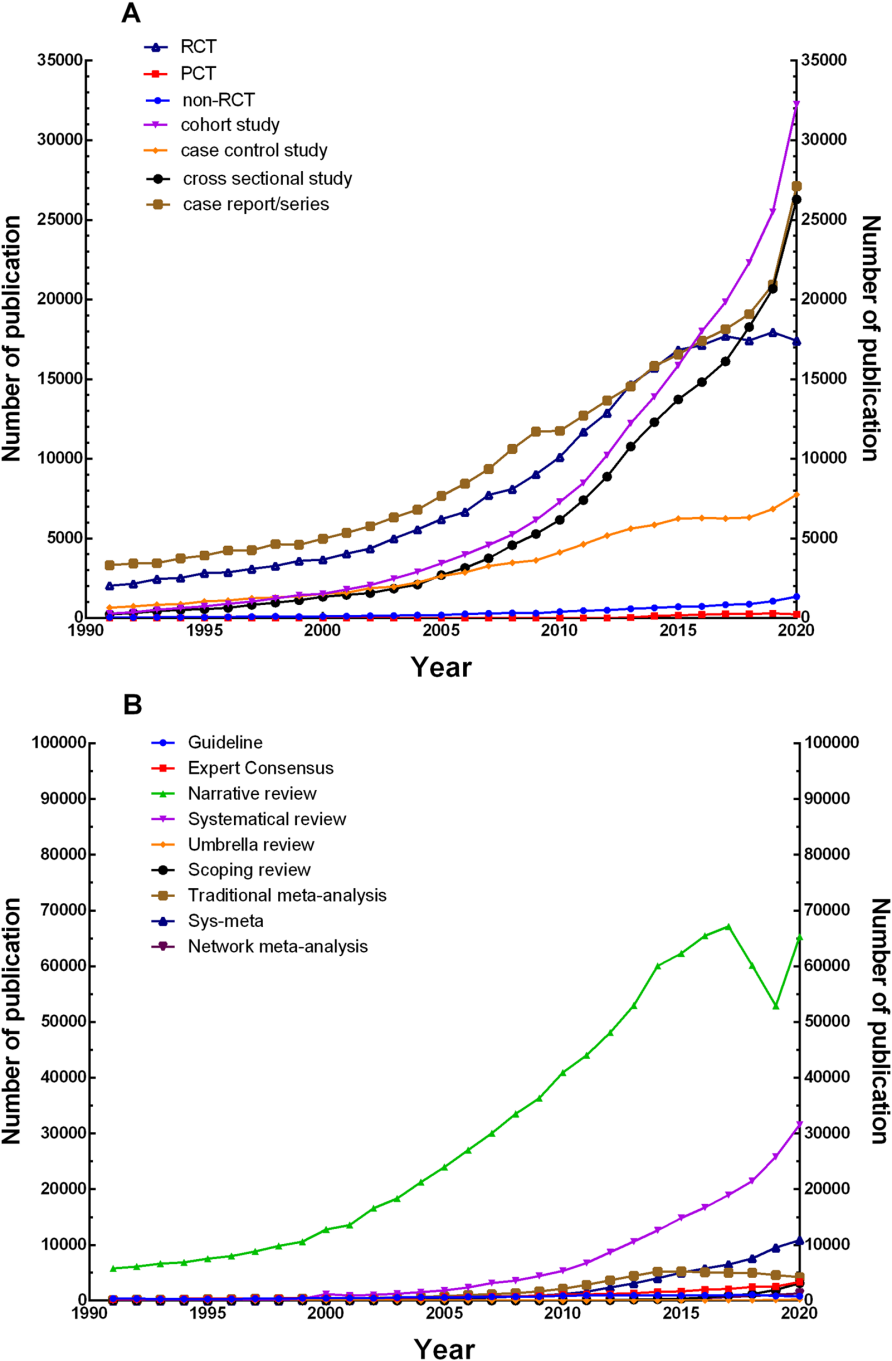
Quantity change trend of each study type in clinical research literature on PubMed database from 1991 to 2020. **A** Trends in the number of primary literature; **B** trends in the number of secondary literature. *RCT* randomized clinical trial; *PCT* pragmatic clinical trial; *Sys*-*meta* systematic review and meta-analysis

Correspondingly, regarding the quantity of publications of secondary literature, the total number of narrative review reached 5,790 in 1991, followed by guidelines with 366, expert consensus with 260, traditional meta-analysis with 139, systematic review with only 8, while network meta-analysis, umbrella review, and scoping review were not yet available. The number of systematic review reached 1,168 in 2000, surpassing guidelines, expert consensus, and traditional meta-analysis for the first time, and has been the top two study type of secondary literature after narrative review ever since. In the year 2002, the number of traditional meta-analysis surpassed guidelines and expert consensus, and became the third most numerous study type of secondary literature until 2016. Systematic review and meta-analysis first appeared in 1994, surpassed guidelines and expert consensus in 2008 and 2009, and traditional meta-analysis for the first time in 2016, becoming the most numerous type of meta-analysis and the third most numerous type of secondary literature (maintaining this to date). Scoping review, network meta-analysis, and umbrella review first appeared in 1999, 2002, and 2006, respectively, and the quantity of publications of such new study types has grown rapidly, reaching 3,119, 1,305, and 222 in 2020, respectively (Fig. 2).

**Fig. 3:**
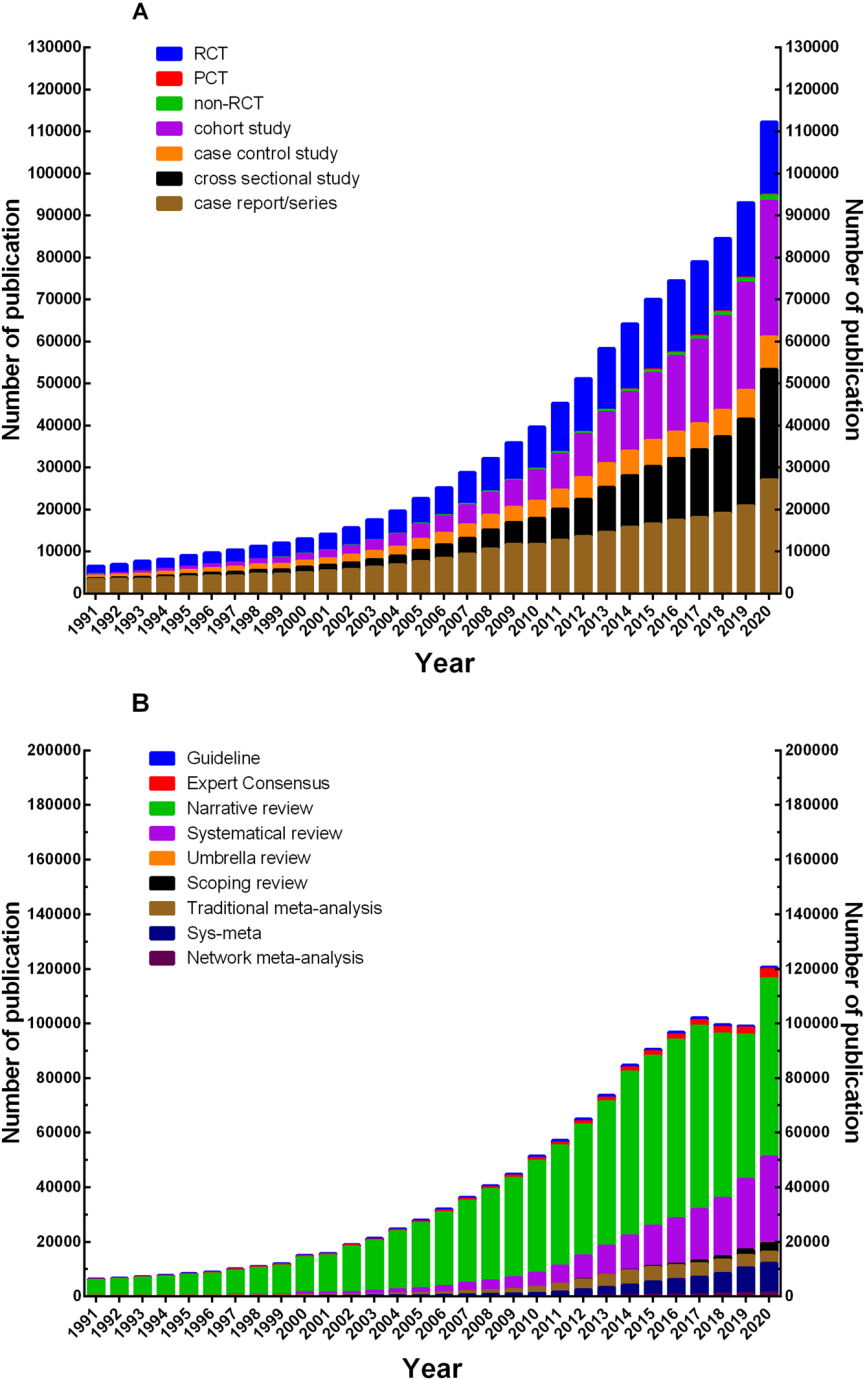
Composition change trend of each research type in clinical research literature in PubMed database from 1991 to 2020. **A** Trend change of primary literature proportion; **B** trend change of secondary literature proportion. *RCT*: randomized clinical trial; *PCT*: pragmatic clinical trial; *Sys*-*meta*: systematic review and meta-analysis

**Fig. 4:**
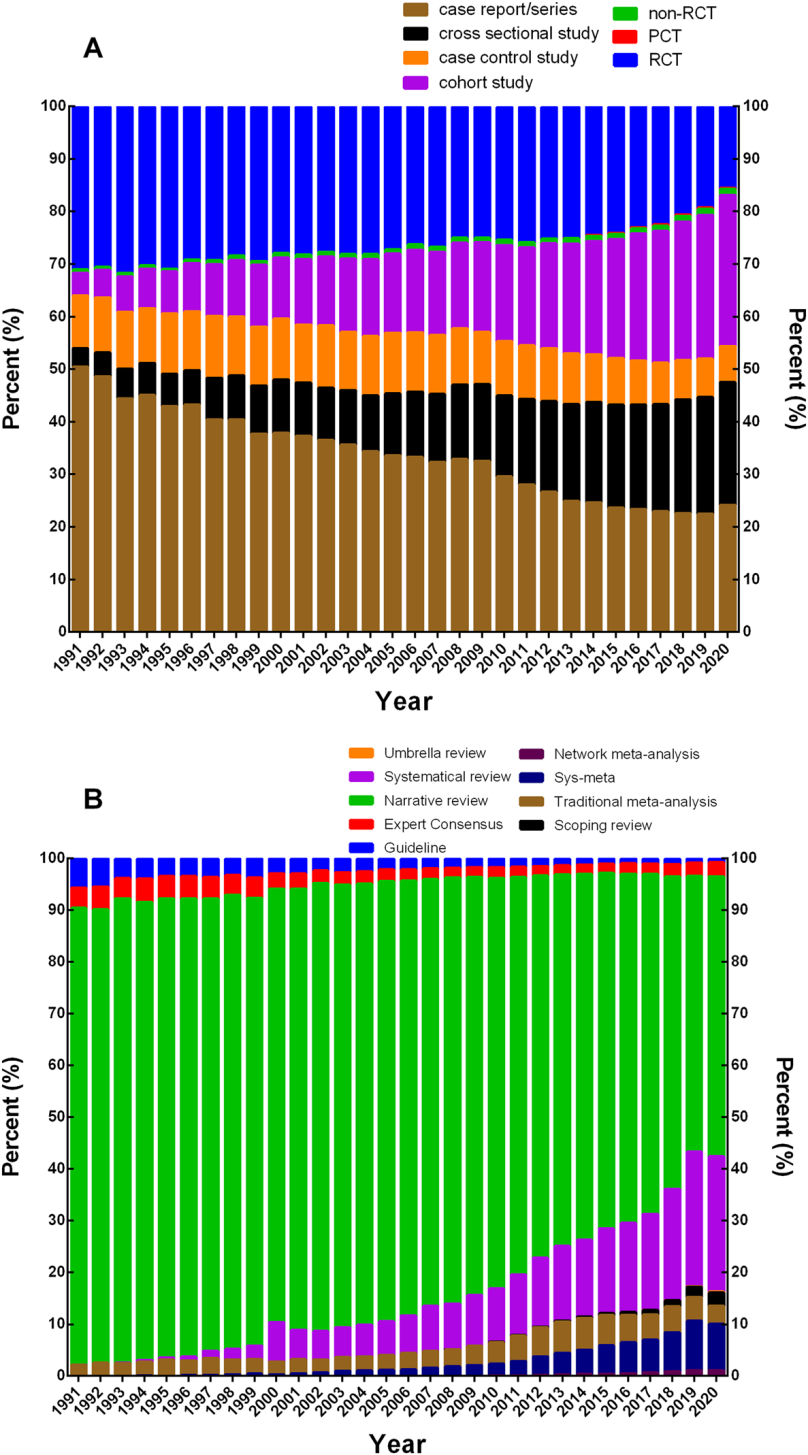
Trend chart of percentage change of each research type in clinical research literature in PubMed database from 1991 to 2020. **A** Trend change of primary literature proportion; **B** trend change of secondary literature proportion. *RCT*: randomized clinical trial; *PCT*: pragmatic clinical trial; *Sys*-*meta*: systematic review and meta-analysis

Regarding the composition ratio of primary literature, the proportion of case report/series and RCTs among primary literature tended to decrease (*P* < 0.05). Conversely, the proportion of cross-sectional study and cohort study had a tendency to increase (*P* < 0.05). Among secondary literature, the proportion of guidelines and narrative review tended to decrease year by year, with the proportion of systematic review, and systematic review and meta-analysis tending to increase (*P* < 0.05) (Figs. 3, 4).

As for the average annual growth rate, the primary literature was 10.28%, ranked from high to low as PCT (83.68%), cohort study (17.74%), cross-sectional study (17.61%), non-RCT (12.11%), case control study (8.86%), RCT (7.68%), and case report/series (7.51%); while the secondary literature was 10.57%, ranked from high to low as network meta-analysis (48.97%), umbrella review (47.09%), scoping review (41.92%), systematic review and meta-analysis (33.44%), systematic review (33.05%), traditional meta-analysis (12.49%), expert consensus (9.22%), narrative review (8.72%), and guidelines (2.82%).

In the primary literature, cross-sectional study and cohort study maintained a growth trend (*P* < 0.05), and the annual growth rate increased steadily in the last 3 years. The annual growth rate of RCT maintained a trend before 2018, but there has been a slight fluctuation in the most recent 3 years. The growth rate of non-RCT, case report/series in 2020 was significantly higher than earlier (*P* < 0.05).

In the secondary literature, the change of the annual growth rate of network meta-analysis, umbrella review, and scoping review was large (*P* < 0.05), while that of annual growth rate of other secondary literature research types remained relatively stable (*P* > 0.05). The annual growth rate of guidelines, expert consensus, and narrative review was relatively lower; while both the systematic review and meta-analysis maintained a stable growth trend (Additional file 1: Tables S3–4, Fig. 4).

**Fig. 5:**
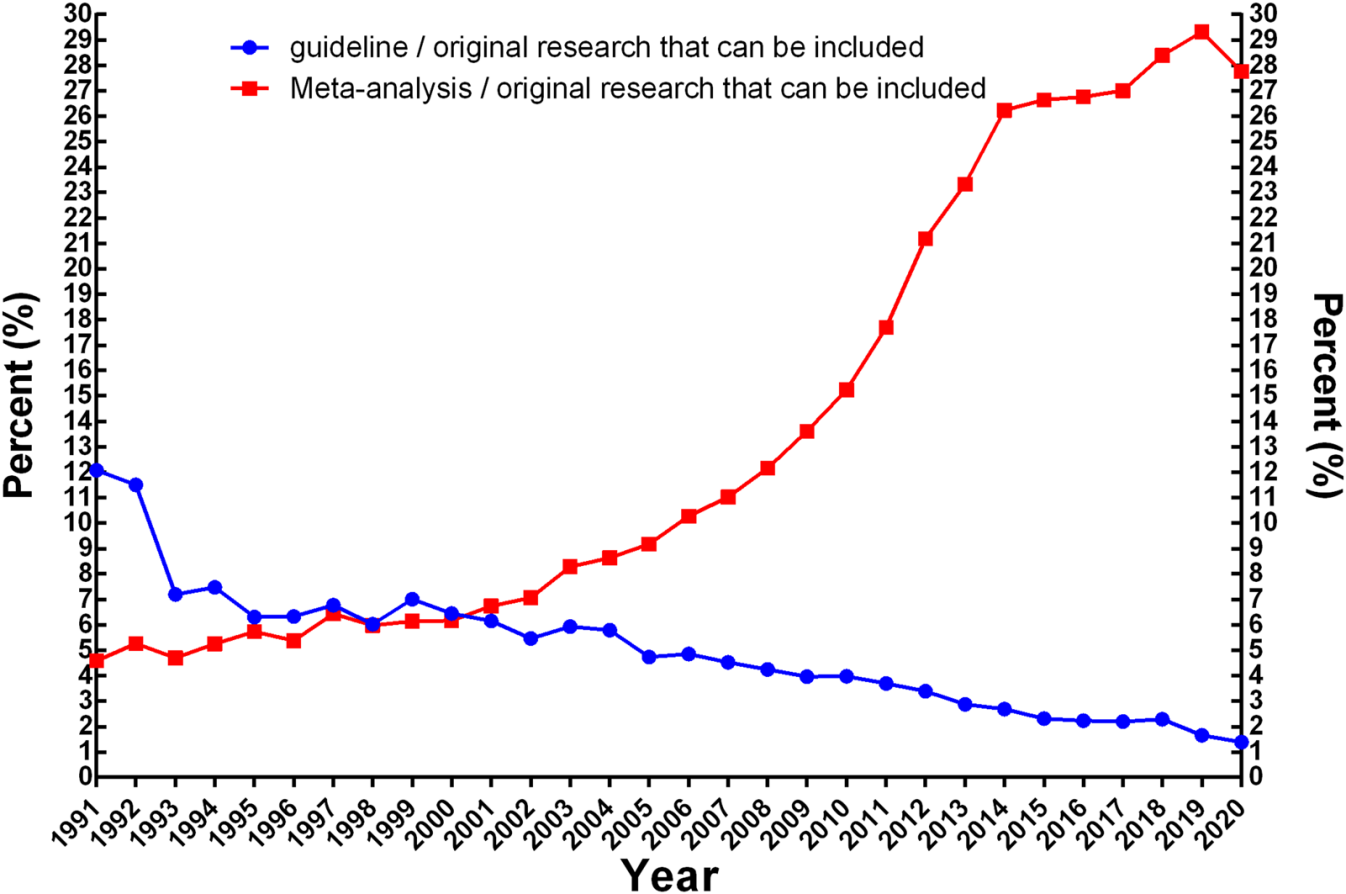
Trend chart of growth rate between secondary literature and primary literature in PubMed database from 1991 to 2020. Blue: the ratio of guideline to the original study; red: the ratio of meta-analysis to the original study

**Fig. 6:**
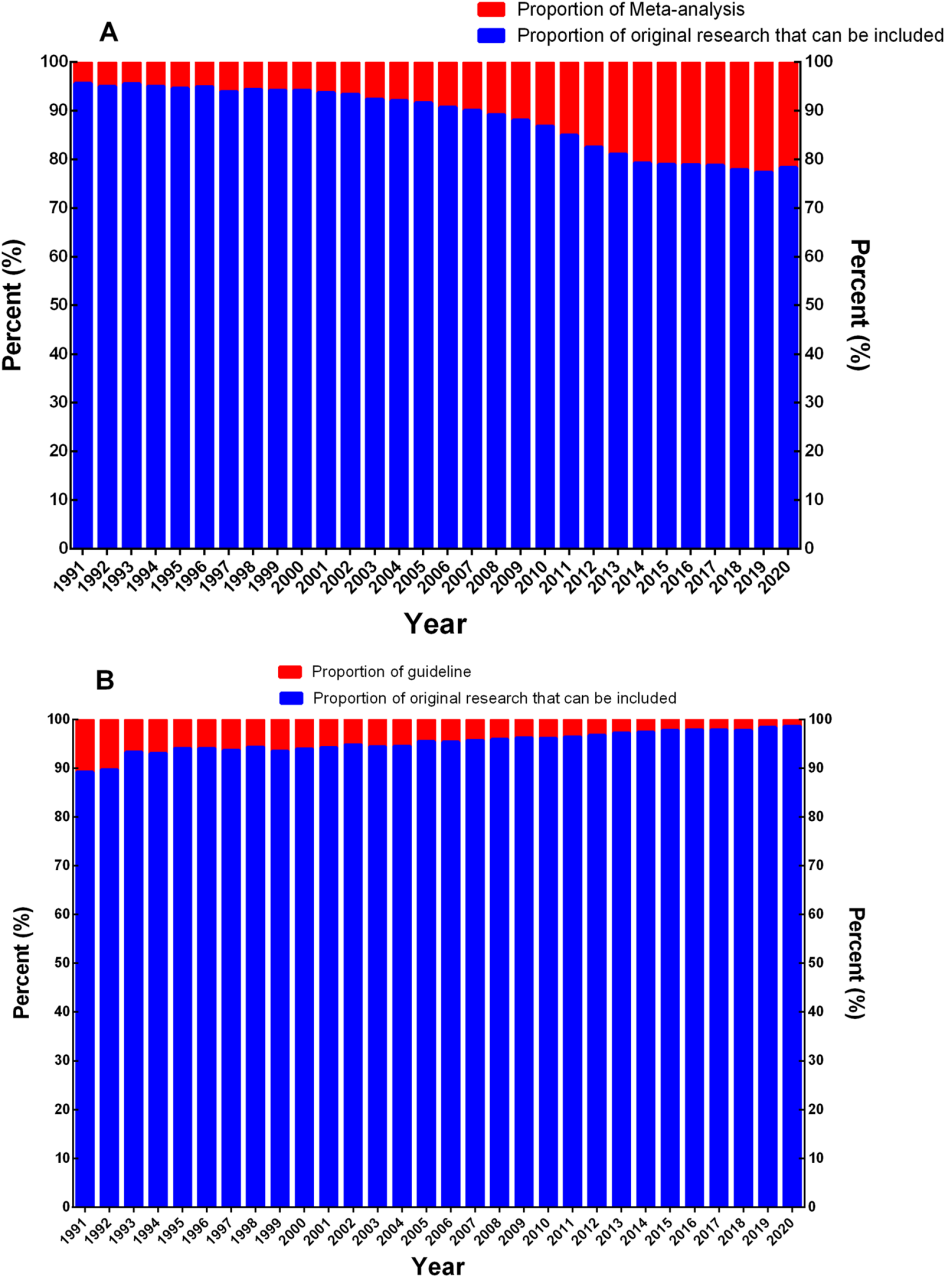
Trend chart of proportion change between secondary literature and primary literature in clinical research literature in PubMed database from 1991 to 2020. Blue: the ratio of guideline to the original study; red: the ratio of meta-analysis to the original study

Based on the analysis of the ratio of secondary literature to the number of original studies, the proportion of all guidelines and meta-analysis to the original studies that were included from 1991 to 2020 were 3.28% and 21.04%, respectively, and the average annual growth rates of the included original studies, guidelines, and meta-analysis that were included were 10.78%, 2.82%, and 17.87%, respectively. Specifically, the ratio of guidelines to primary literature significantly decreased (*P* < 0.05), from 12.08% in 1991 to 1.39% in 2020, with a generally stable decline rate; while the proportion of meta-analysis and included primary literature increased year by year, from 4.59% in 1991 to 27.76% in 2020 (*P* < 0.05), of which the increase was at its highest from 2008 to 2014, after which the growth rate stabilized (Figs. 5, 6).

## Discussion

PubMed is the most widely used free database in the field of medical health. In the last 30 years, the increase of scientific researchers, and the update of scientific research methods, the number of papers and research design has exploded. This study extracted the number of publications of different research types published on PubMed for the last three decades, and defined the change in trend of the number of publications of different research designs during this period by analyzing the change in trend of their number, growth rate, composition ratio, and the proportional relationship between primary literature and secondary literature research. Combined with the quality level of EBM corresponding to different research designs [23–36], this study provides researchers with an overview of different research design publications for the 30 years under study and provides a change trend of medical research.

In the original studies, case report/series (as the research type with the largest number of early studies), have a low average annual growth rate, and their proportion in the original studies decreased year by year. At present, they are no longer the primary literature type with the largest proportion. Although the sum of the proportion of cohort study and cross-sectional study was only about 8% in 1991, their average annual growth rate reached about 18%. Currently, the proportion of these types has surpassed that of RCT and become one of the largest types of primary literature.

The number of RCTs increased steadily from 1991 to 2017, its proportion in the original studies ranked second from 1991 to 2014. The number of RCTs surpassed case report/series for the first time in 2015 but fell to the fourth place from 2016 to 2020, which may be related to the standardization and high ethical requirements of RCT [23].

As a control trial closer to the application in the real world, PCT firstly appeared in 2011 [24] PCT currently accounts for a low proportion of primary literature. Considering its strong clinical application and the advantages that the research design basically follows the control experiment, we think PCT may attract more researchers’ attention in the future.

In the secondary literature research, the narrative review accounted for 88%, while the guidelines and expert consensus accounted for nearly 10% in 1991. Later, the average annual growth rates of narrative review and guidelines became the lowest in secondary literature research, and their proportion also decreased year by year. Currently, although narrative review is still the secondary literature with the highest proportion has been greatly reduced compared with 1991, from more than 85% to less than 20% at present. The proportion of guidelines has even fallen below 1%, and we believe this trend will develop further in the future.

Compared with narrative review, systematic review and meta-analysis show a significantly growth rate. Combined with the fact that systematic review and meta-analysis are higher than narrative review in the level of evidence quality [9–13], we believe that this trend will be further deepened in the future.

Scoping review [25], which can describe the research results and research scope of a specific research field in more detail by investigating or exploring the research status, degree, and methodology of a research field or topic, appeared for the first time in 1999. Network meta-analysis [26] can analyze the relationship between more than two interventions based on multiple studies by means of indirect comparison or mixed comparison, appeared for the first time in 2002; while in 2006, the umbrella review [27] appeared to summarize broader evidence when the systematic review and meta-analysis of a medical research topic reached a certain number. These new research types increased rapidly, with an average annual growth rate of more than 40%, further enriching the methods of secondary literature research.

Our research shows that the average annual growth rate of meta-analysis (17.87%) from 1991 to 2020 is significantly higher than that of included primary literature (10.78%) and guidelines (2.82%). The proportion of guidelines to included primary literature decreased year by year, from 12.08% in 1991 to 1.39% in 2020, while the proportion of meta-analysis to primary literature increased year by year, from 4.59% in 1991 to 27.76% in 2020. For the ratio between meta-analysis and primary literature, each meta-analysis needs to include a certain amount of primary literature to ensure the robustness of research results of meta-analysis. However, there were many overlapped and partially overlapped meta-analyses, which means that the meta-analysis with high quality and high evidence level is reduced. Therefore, we suggest that attention paid to meta-analysis be reduced and primary literature strengthened, especially RCT with its high evidence level [9–12].

Compared with other studies focusing on the characteristics of literature related to specific diseases [20], our study is the first to analyze the changes in the number, growth rate, composition ratio, and the proportional relationship between the primary literature and the secondary literature of all clinical original studies and secondary literature studies against the background of the explosive growth in the number of various research methods and publications in the last 30 years. This study defined the classification characteristics and change trend of clinical research literature, and provided researchers with an overview of different research design publications in recent 30 years, as well as a reference for the rational allocation of research resources in future.

Our research also has some limitations. Firstly, we only extracted the number of publications of different research types without evaluating their quality. Secondly, the classification of this document did not not really represent the actual type of research, because some records in Pubmed database are vague. Thirdly, some literatures may overlap, which may cause information bias. Finally, we only used the PubMed database for literature retrieval, and the retrieval was dependent on the use of keywords and PubMed filtering tools. Hence, there is a risk of retrieval bias [37] in the study.

## Conclusions

The characteristics of clinical research literature have changed significantly from 1991 to 2020. Based on the trend, the case report/series, case control study, and narrative review are on the decline, while cohort study, cross-sectional study, systematic reviews, and systematic review and meta-analysis literature have increased. To improve the quality of clinical evidence, we recommend RCT and cohort study give priority to access to allocated research resources in future.

## Supplementary Information

**Additional file 1: Table S1. **Statistical data of literatures for original studies. **Table S2.** Statistical data of secondary literatures. **Table S3.** Growth rate of original studies. **Table S4.** Growth rate of secondary literatures.

## Data Availability

Not applicable.

## Abbreviations

RCT: Randomized clinical trial
PCT: Pragmatic clinical trial
NCBI: The National Center for Biotechnology Information
NLM: The National Library of Medicine
EBM: Evidence-based medicine
GRADE: Grading of recommendations, assessment, development and evaluation
