# Supplementary material for: Changing trends in clinical research literature on PubMed database from 1991 to 2020
Source: Eur J Med Res. 2022 Jun 20;27:95. doi: 10.1186/s40001-022-00717-9 (PMC9208110; doi:10.1186/s40001-022-00717-9)
Supplement: Supplementary file 1 — Additional file 1: Table S1. Statistical data of literatures for original studies. Table S2. Statistical data of secondary literatures. Table S3. Growth rate of original studies. Table S4. Growth rate of secondary literatures. [file 40001_2022_717_MOESM1_ESM.docx]

**Additional file materials**

**Additional file Table S1. Statistical data of literatures for original studies**

| **Year** | **RCT** | | **PCT** | | **Non-RCT** | | **Cohort study** | | **Case control study** | | **Cross sectional study** | | **Case report/series** | | **total** |
| --- | --- | --- | --- | --- | --- | --- | --- | --- | --- | --- | --- | --- | --- | --- | --- |
|  | **N** | **%** | **N** | **%** | **N** | **%** | **N** | **%** | **N** | **%** | **N** | **%** | **N** | **%** |  |
| 1991 | 2037 | 30.92% | 0 | 0.00% | 49 | 0.74% | 283 | 4.30% | 661 | 10.03% | 238 | 3.61% | 3320 | 50.39% | 6588 |
| 1992 | 2155 | 30.55% | 0 | 0.00% | 40 | 0.57% | 370 | 5.25% | 740 | 10.49% | 320 | 4.54% | 3428 | 48.60% | 7053 |
| 1993 | 2457 | 31.66% | 0 | 0.00% | 49 | 0.63% | 527 | 6.79% | 843 | 10.86% | 439 | 5.66% | 3445 | 44.39% | 7760 |
| 1994 | 2522 | 30.21% | 0 | 0.00% | 55 | 0.66% | 630 | 7.55% | 872 | 10.45% | 511 | 6.12% | 3757 | 45.01% | 8347 |
| 1995 | 2825 | 30.87% | 0 | 0.00% | 48 | 0.52% | 735 | 8.03% | 1049 | 11.46% | 566 | 6.19% | 3927 | 42.92% | 9150 |
| 1996 | 2872 | 29.16% | 0 | 0.00% | 70 | 0.71% | 902 | 9.16% | 1104 | 11.21% | 647 | 6.57% | 4253 | 43.19% | 9848 |
| 1997 | 3088 | 29.27% | 0 | 0.00% | 82 | 0.78% | 1036 | 9.82% | 1247 | 11.82% | 834 | 7.90% | 4264 | 40.41% | 10551 |
| 1998 | 3271 | 28.40% | 0 | 0.00% | 105 | 0.91% | 1235 | 10.72% | 1289 | 11.19% | 970 | 8.42% | 4649 | 40.36% | 11519 |
| 1999 | 3592 | 29.40% | 0 | 0.00% | 91 | 0.74% | 1439 | 11.78% | 1372 | 11.23% | 1119 | 9.16% | 4603 | 37.68% | 12216 |
| 2000 | 3667 | 27.88% | 0 | 0.00% | 114 | 0.87% | 1527 | 11.61% | 1528 | 11.62% | 1335 | 10.15% | 4981 | 37.87% | 13152 |
| 2001 | 4040 | 28.16% | 0 | 0.00% | 127 | 0.89% | 1786 | 12.45% | 1586 | 11.06% | 1459 | 10.17% | 5347 | 37.27% | 14345 |
| 2002 | 4369 | 27.65% | 0 | 0.00% | 145 | 0.92% | 2074 | 13.13% | 1879 | 11.89% | 1568 | 9.92% | 5766 | 36.49% | 15801 |
| 2003 | 4992 | 28.12% | 0 | 0.00% | 158 | 0.89% | 2475 | 13.94% | 1965 | 11.07% | 1848 | 10.41% | 6317 | 35.58% | 17755 |
| 2004 | 5563 | 28.08% | 0 | 0.00% | 197 | 0.99% | 2906 | 14.67% | 2228 | 11.25% | 2108 | 10.64% | 6806 | 34.36% | 19808 |
| 2005 | 6212 | 27.18% | 0 | 0.00% | 195 | 0.85% | 3441 | 15.06% | 2643 | 11.57% | 2693 | 11.79% | 7667 | 33.55% | 22851 |
| 2006 | 6666 | 26.28% | 0 | 0.00% | 256 | 1.01% | 3988 | 15.72% | 2866 | 11.30% | 3148 | 12.41% | 8437 | 33.27% | 25361 |
| 2007 | 7729 | 26.67% | 0 | 0.00% | 296 | 1.02% | 4584 | 15.82% | 3259 | 11.25% | 3752 | 12.95% | 9357 | 32.29% | 28977 |
| 2008 | 8097 | 25.04% | 0 | 0.00% | 318 | 0.98% | 5250 | 16.24% | 3474 | 10.74% | 4578 | 14.16% | 10618 | 32.84% | 32335 |
| 2009 | 9026 | 25.00% | 0 | 0.00% | 311 | 0.86% | 6156 | 17.05% | 3622 | 10.03% | 5279 | 14.62% | 11713 | 32.44% | 36107 |
| 2010 | 10111 | 25.38% | 0 | 0.00% | 400 | 1.00% | 7285 | 18.29% | 4121 | 10.35% | 6159 | 15.46% | 11758 | 29.52% | 39834 |
| 2011 | 11694 | 25.77% | 1 | 0.00% | 465 | 1.02% | 8489 | 18.71% | 4628 | 10.20% | 7399 | 16.31% | 12700 | 27.99% | 45376 |
| 2012 | 12883 | 25.10% | 2 | 0.00% | 495 | 0.96% | 10237 | 19.95% | 5179 | 10.09% | 8872 | 17.29% | 13652 | 26.60% | 51320 |
| 2013 | 14661 | 25.08% | 41 | 0.07% | 590 | 1.01% | 12228 | 20.92% | 5615 | 9.61% | 10771 | 18.43% | 14551 | 24.89% | 58457 |
| 2014 | 15712 | 24.41% | 129 | 0.20% | 640 | 0.99% | 13898 | 21.59% | 5856 | 9.10% | 12303 | 19.11% | 15827 | 24.59% | 64365 |
| 2015 | 16832 | 24.01% | 178 | 0.25% | 711 | 1.01% | 15855 | 22.62% | 6246 | 8.91% | 13728 | 19.59% | 16542 | 23.60% | 70092 |
| 2016 | 17140 | 22.98% | 211 | 0.28% | 733 | 0.98% | 18004 | 24.13% | 6282 | 8.42% | 14817 | 19.86% | 17414 | 23.34% | 74601 |
| 2017 | 17700 | 22.37% | 258 | 0.33% | 838 | 1.06% | 19834 | 25.06% | 6252 | 7.90% | 16117 | 20.37% | 18132 | 22.91% | 79131 |
| 2018 | 17425 | 20.61% | 265 | 0.31% | 879 | 1.04% | 22321 | 26.40% | 6309 | 7.46% | 18270 | 21.61% | 19086 | 22.57% | 84555 |
| 2019 | 17945 | 19.24% | 297 | 0.32% | 1065 | 1.14% | 25495 | 27.34% | 6849 | 7.35% | 20670 | 22.17% | 20925 | 22.44% | 93246 |
| 2020 | 17415 | 15.49% | 238 | 0.21% | 1349 | 1.20% | 32247 | 28.69% | 7750 | 6.89% | 26283 | 23.38% | 27123 | 24.13% | 112405 |
| Total | 254698 | 23.62% | 1620 | 0.15% | 10871 | 1.01% | 226954 | 21.05% | 98653 | 9.15% | 188563 | 17.49% | 297045 | 27.54% | 1078404 |

**RCT: randomized clinical trial; PCT: pragmatic clinical trial.**

**Additional file Table S2. Statistical data of secondary literatures**

| **Year** | **Guideline** | | **Expert Consensus** | | **Narrative review** | | **Systematical review** | | **Umbrella review** | | **Scoping review** | | **Traditional meta-analysis** | | **Sys-meta** | | **network meta-analysis** | | **total** |
| --- | --- | --- | --- | --- | --- | --- | --- | --- | --- | --- | --- | --- | --- | --- | --- | --- | --- | --- | --- |
|  | **N** | **%** | **N** | **%** | **N** | **%** | **N** | **%** | **N** | **%** | **N** | **%** | **N** | **%** | **N** | **%** | **N** | **%** |  |
| 1991 | 366 | 5.58% | 260 | 3.96% | 5790 | 88.22% | 8 | 0.12% | 0 | 0.00% | 0 | 0.00% | 139 | 2.12% | 0 | 0.00% | 0 | 0.00% | 6563 |
| 1992 | 380 | 5.45% | 312 | 4.47% | 6105 | 87.55% | 2 | 0.03% | 0 | 0.00% | 0 | 0.00% | 174 | 2.50% | 0 | 0.00% | 0 | 0.00% | 6973 |
| 1993 | 279 | 3.77% | 293 | 3.96% | 6645 | 89.71% | 8 | 0.11% | 0 | 0.00% | 0 | 0.00% | 182 | 2.46% | 0 | 0.00% | 0 | 0.00% | 7407 |
| 1994 | 305 | 3.93% | 349 | 4.50% | 6861 | 88.46% | 27 | 0.35% | 0 | 0.00% | 0 | 0.00% | 208 | 2.68% | 6 | 0.08% | 0 | 0.00% | 7756 |
| 1995 | 294 | 3.46% | 364 | 4.29% | 7530 | 88.66% | 38 | 0.45% | 0 | 0.00% | 0 | 0.00% | 265 | 3.12% | 2 | 0.02% | 0 | 0.00% | 8493 |
| 1996 | 313 | 3.47% | 395 | 4.37% | 7990 | 88.46% | 68 | 0.75% | 0 | 0.00% | 0 | 0.00% | 257 | 2.85% | 9 | 0.10% | 0 | 0.00% | 9032 |
| 1997 | 369 | 3.65% | 417 | 4.13% | 8825 | 87.36% | 140 | 1.39% | 0 | 0.00% | 0 | 0.00% | 334 | 3.31% | 17 | 0.17% | 0 | 0.00% | 10102 |
| 1998 | 356 | 3.18% | 430 | 3.84% | 9824 | 87.77% | 230 | 2.05% | 0 | 0.00% | 0 | 0.00% | 326 | 2.91% | 27 | 0.24% | 0 | 0.00% | 11193 |
| 1999 | 455 | 3.72% | 477 | 3.90% | 10584 | 86.54% | 313 | 2.56% | 0 | 0.00% | 2 | 0.02% | 346 | 2.83% | 53 | 0.43% | 0 | 0.00% | 12230 |
| 2000 | 441 | 2.89% | 455 | 2.98% | 12769 | 83.68% | 1168 | 7.65% | 0 | 0.00% | 6 | 0.04% | 376 | 2.46% | 45 | 0.29% | 0 | 0.00% | 15260 |
| 2001 | 464 | 2.92% | 467 | 2.94% | 13556 | 85.24% | 908 | 5.71% | 0 | 0.00% | 1 | 0.01% | 434 | 2.73% | 74 | 0.47% | 0 | 0.00% | 15904 |
| 2002 | 462 | 2.41% | 464 | 2.42% | 16591 | 86.53% | 1057 | 5.51% | 0 | 0.00% | 1 | 0.01% | 477 | 2.49% | 121 | 0.63% | 1 | 0.01% | 19174 |
| 2003 | 569 | 2.65% | 524 | 2.44% | 18348 | 85.43% | 1240 | 5.77% | 0 | 0.00% | 3 | 0.01% | 582 | 2.71% | 211 | 0.98% | 1 | 0.00% | 21478 |
| 2004 | 631 | 2.53% | 589 | 2.36% | 21270 | 85.19% | 1534 | 6.14% | 0 | 0.00% | 2 | 0.01% | 688 | 2.76% | 252 | 1.01% | 1 | 0.00% | 24967 |
| 2005 | 592 | 2.10% | 630 | 2.24% | 23961 | 85.02% | 1850 | 6.56% | 0 | 0.00% | 6 | 0.02% | 819 | 2.91% | 325 | 1.15% | 1 | 0.00% | 28184 |
| 2006 | 668 | 2.08% | 704 | 2.19% | 27030 | 83.97% | 2364 | 7.34% | 1 | 0.00% | 8 | 0.02% | 1022 | 3.17% | 393 | 1.22% | 1 | 0.00% | 32191 |
| 2007 | 719 | 1.97% | 767 | 2.10% | 30028 | 82.41% | 3165 | 8.69% | 1 | 0.00% | 10 | 0.03% | 1186 | 3.25% | 557 | 1.53% | 5 | 0.01% | 36438 |
| 2008 | 726 | 1.78% | 787 | 1.93% | 33511 | 82.27% | 3611 | 8.86% | 1 | 0.00% | 15 | 0.04% | 1331 | 3.27% | 744 | 1.83% | 9 | 0.02% | 40735 |
| 2009 | 757 | 1.68% | 885 | 1.96% | 36348 | 80.69% | 4426 | 9.83% | 3 | 0.01% | 26 | 0.06% | 1678 | 3.73% | 915 | 2.03% | 8 | 0.02% | 45046 |
| 2010 | 873 | 1.69% | 1090 | 2.11% | 40953 | 79.27% | 5351 | 10.36% | 1 | 0.00% | 51 | 0.10% | 2148 | 4.16% | 1170 | 2.26% | 23 | 0.04% | 51660 |
| 2011 | 933 | 1.63% | 1149 | 2.00% | 44016 | 76.71% | 6743 | 11.75% | 4 | 0.01% | 62 | 0.11% | 2830 | 4.93% | 1595 | 2.78% | 49 | 0.09% | 57381 |
| 2012 | 975 | 1.50% | 1206 | 1.85% | 48107 | 73.81% | 8686 | 13.33% | 5 | 0.01% | 96 | 0.15% | 3646 | 5.59% | 2360 | 3.62% | 96 | 0.15% | 65177 |
| 2013 | 950 | 1.29% | 1356 | 1.84% | 52971 | 71.76% | 10632 | 14.40% | 4 | 0.01% | 172 | 0.23% | 4469 | 6.05% | 3111 | 4.21% | 150 | 0.20% | 73815 |
| 2014 | 973 | 1.14% | 1571 | 1.85% | 60057 | 70.67% | 12623 | 14.85% | 7 | 0.01% | 249 | 0.29% | 5225 | 6.15% | 4024 | 4.73% | 257 | 0.30% | 84986 |
| 2015 | 918 | 1.01% | 1683 | 1.85% | 62312 | 68.67% | 14837 | 16.35% | 20 | 0.02% | 362 | 0.40% | 5262 | 5.80% | 5006 | 5.52% | 341 | 0.38% | 90741 |
| 2016 | 944 | 0.97% | 1977 | 2.04% | 65444 | 67.45% | 16703 | 17.22% | 37 | 0.04% | 579 | 0.60% | 5092 | 5.25% | 5792 | 5.97% | 456 | 0.47% | 97024 |
| 2017 | 987 | 0.96% | 2109 | 2.06% | 67157 | 65.63% | 18972 | 18.54% | 53 | 0.05% | 930 | 0.91% | 4983 | 4.87% | 6489 | 6.34% | 650 | 0.64% | 102330 |
| 2018 | 1081 | 1.08% | 2472 | 2.48% | 60160 | 60.28% | 21445 | 21.49% | 77 | 0.08% | 1163 | 1.17% | 5002 | 5.01% | 7565 | 7.58% | 832 | 0.83% | 99797 |
| 2019 | 859 | 0.86% | 2547 | 2.56% | 52892 | 53.25% | 25816 | 25.99% | 129 | 0.13% | 1943 | 1.96% | 4586 | 4.62% | 9527 | 9.59% | 1030 | 1.04% | 99329 |
| 2020 | 820 | 0.68% | 3355 | 2.78% | 65338 | 54.08% | 31576 | 26.14% | 222 | 0.18% | 3119 | 2.58% | 4216 | 3.49% | 10856 | 8.99% | 1305 | 1.08% | 120807 |
| Total | 19459 | 1.49% | 30084 | 2.31% | 922973 | 70.88% | 195541 | 15.02% | 565 | 0.04% | 8806 | 0.68% | 58283 | 4.48% | 61246 | 4.70% | 5216 | 0.40% | 1302173 |

Sys-meta: systematic review and meta-analysis.

**Additional file Table S3. Growth rate of original studies**

| **Year** | **RCT** | **PCT** | **Non-RCT** | **Cohort study** | **Case control study** | **Cross sectional study** | **Case report/series** | **Total** | **The included original researches of Guideline/Meta analysis*** |
| --- | --- | --- | --- | --- | --- | --- | --- | --- | --- |
| 1992 | 5.79% | - | -18.37% | 30.74% | 11.95% | 34.45% | 3.25% | 7.06% | 9.08% |
| 1993 | 14.01% | - | 22.50% | 42.43% | 13.92% | 37.19% | 0.50% | 10.02% | 17.28% |
| 1994 | 2.65% | - | 12.24% | 19.54% | 3.44% | 16.40% | 9.06% | 7.56% | 5.24% |
| 1995 | 12.01% | - | -12.73% | 16.67% | 20.30% | 10.76% | 4.52% | 9.62% | 14.17% |
| 1996 | 1.66% | - | 45.83% | 22.72% | 5.24% | 14.31% | 8.30% | 7.63% | 6.25% |
| 1997 | 7.52% | - | 17.14% | 14.86% | 12.95% | 28.90% | 0.26% | 7.14% | 10.21% |
| 1998 | 5.93% | - | 28.05% | 19.21% | 3.37% | 16.31% | 9.03% | 9.17% | 8.20% |
| 1999 | 9.81% | - | -13.33% | 16.52% | 6.44% | 15.36% | -0.99% | 6.05% | 10.07% |
| 2000 | 2.09% | - | 25.27% | 6.12% | 11.37% | 19.30% | 8.21% | 7.66% | 5.27% |
| 2001 | 10.17% | - | 11.40% | 16.96% | 3.80% | 9.29% | 7.35% | 9.07% | 10.28% |
| 2002 | 8.14% | - | 14.17% | 16.13% | 18.47% | 7.47% | 7.84% | 10.15% | 12.31% |
| 2003 | 14.26% | - | 8.97% | 19.33% | 4.58% | 17.86% | 9.56% | 12.37% | 13.26% |
| 2004 | 11.44% | - | 24.68% | 17.41% | 13.38% | 14.07% | 7.74% | 11.56% | 13.60% |
| 2005 | 11.67% | - | -1.02% | 18.41% | 18.63% | 27.75% | 12.65% | 15.36% | 14.66% |
| 2006 | 7.31% | - | 31.28% | 15.90% | 8.44% | 16.90% | 10.04% | 10.98% | 10.29% |
| 2007 | 15.95% | - | 15.63% | 14.94% | 13.71% | 19.19% | 10.90% | 14.26% | 15.19% |
| 2008 | 4.76% | - | 7.43% | 14.53% | 6.60% | 22.01% | 13.48% | 11.59% | 8.01% |
| 2009 | 11.47% | - | -2.20% | 17.26% | 4.26% | 15.31% | 10.31% | 11.67% | 11.53% |
| 2010 | 12.02% | - | 28.62% | 18.34% | 13.78% | 16.67% | 0.38% | 10.32% | 14.66% |
| 2011 | 15.66% | - | 16.25% | 16.53% | 12.30% | 20.13% | 8.01% | 13.91% | 15.33% |
| 2012 | 10.17% | 100.00% | 6.45% | 20.59% | 11.91% | 19.91% | 7.50% | 13.10% | 13.92% |
| 2013 | 13.80% | 1950.00% | 19.19% | 19.45% | 8.42% | 21.40% | 6.59% | 13.91% | 15.07% |
| 2014 | 7.17% | 214.63% | 8.47% | 13.66% | 4.29% | 14.22% | 8.77% | 10.11% | 9.36% |
| 2015 | 7.13% | 37.98% | 11.09% | 14.08% | 6.66% | 11.58% | 4.52% | 8.90% | 9.90% |
| 2016 | 1.83% | 18.54% | 3.09% | 13.55% | 0.58% | 7.93% | 5.27% | 6.43% | 6.40% |
| 2017 | 3.27% | 22.27% | 14.32% | 10.16% | -0.48% | 8.77% | 4.12% | 6.07% | 5.93% |
| 2018 | -1.55% | 2.71% | 4.89% | 12.54% | 0.91% | 13.36% | 5.26% | 6.85% | 5.16% |
| 2019 | 2.98% | 12.08% | 21.16% | 14.22% | 8.56% | 13.14% | 9.64% | 10.28% | 9.43% |
| 2020 | -2.95% | -19.87% | 26.67% | 26.48% | 13.16% | 27.16% | 29.62% | 20.55% | 14.23% |
| **Average annual growth rate** | 7.68% | 83.68% | 12.11% | 17.74% | 8.86% | 17.61% | 7.51% | 10.28% | 10.78% |

***The included original researches are made up of RCT, PCT, Non-RCT, cohort study and case control study.**

**Additional file Table S4. Growth rate of secondary literatures**

| **Year** | **Guideline** | **Expert Consensus** | **Narrative review** | **Systematical review** | **Umbrella review** | **Scoping review** | **Traditional meta-analysis** | **Sys-meta** | **network meta-analysis** | **Total** | **Meta-analysis*** |
| --- | --- | --- | --- | --- | --- | --- | --- | --- | --- | --- | --- |
| 1992 | 3.83% | 20.00% | 5.44% | -75.00% | - | - | 25.18% | - | - | 6.25% | 25.18% |
| 1993 | -26.58% | -6.09% | 8.85% | 300.00% | - | - | 4.60% | - | - | 6.22% | 4.60% |
| 1994 | 9.32% | 19.11% | 3.25% | 237.50% | - | - | 14.29% | - | - | 4.71% | 17.58% |
| 1995 | -3.61% | 4.30% | 9.75% | 40.74% | - | - | 27.40% | -66.67% | - | 9.50% | 24.77% |
| 1996 | 6.46% | 8.52% | 6.11% | 78.95% | - | - | -3.02% | 350.00% | - | 6.35% | -0.37% |
| 1997 | 17.89% | 5.57% | 10.45% | 105.88% | - | - | 29.96% | 88.89% | - | 11.85% | 31.95% |
| 1998 | -3.52% | 3.12% | 11.32% | 64.29% | - | - | -2.40% | 58.82% | - | 10.80% | 0.57% |
| 1999 | 27.81% | 10.93% | 7.74% | 36.09% | - | - | 6.13% | 96.30% | - | 9.26% | 13.03% |
| 2000 | -3.08% | -4.61% | 20.64% | 273.16% | - | 200.00% | 8.67% | -15.09% | - | 24.78% | 5.51% |
| 2001 | 5.22% | 2.64% | 6.16% | -22.26% | - | -83.33% | 15.43% | 64.44% | - | 4.22% | 20.67% |
| 2002 | -0.43% | -0.64% | 22.39% | 16.41% | - | 0.00% | 9.91% | 63.51% | - | 20.56% | 17.91% |
| 2003 | 23.16% | 12.93% | 10.59% | 17.31% | - | 200.00% | 22.01% | 74.38% | 0.00% | 12.02% | 32.55% |
| 2004 | 10.90% | 12.40% | 15.93% | 23.71% | - | -33.33% | 18.21% | 19.43% | 0.00% | 16.24% | 18.51% |
| 2005 | -6.18% | 6.96% | 12.65% | 20.60% | - | 200.00% | 19.04% | 28.97% | 0.00% | 12.89% | 21.68% |
| 2006 | 12.84% | 11.75% | 12.81% | 27.78% | - | 33.33% | 24.79% | 20.92% | 0.00% | 14.22% | 23.67% |
| 2007 | 7.63% | 8.95% | 11.09% | 33.88% | 0.00% | 25.00% | 16.05% | 41.73% | 400.00% | 13.19% | 23.45% |
| 2008 | 0.97% | 2.61% | 11.60% | 14.09% | 0.00% | 50.00% | 12.23% | 33.57% | 80.00% | 11.79% | 19.22% |
| 2009 | 4.27% | 12.45% | 8.47% | 22.57% | 200.00% | 73.33% | 26.07% | 22.98% | -11.11% | 10.58% | 24.81% |
| 2010 | 15.32% | 23.16% | 12.67% | 20.90% | -66.67% | 96.15% | 28.01% | 27.87% | 187.50% | 14.68% | 28.45% |
| 2011 | 6.87% | 5.41% | 7.48% | 26.01% | 300.00% | 21.57% | 31.75% | 36.32% | 113.04% | 11.07% | 33.91% |
| 2012 | 4.50% | 4.96% | 9.29% | 28.82% | 25.00% | 54.84% | 28.83% | 47.96% | 95.92% | 13.59% | 36.39% |
| 2013 | -2.56% | 12.44% | 10.11% | 22.40% | -20.00% | 79.17% | 22.57% | 31.82% | 56.25% | 13.25% | 26.68% |
| 2014 | 2.42% | 15.86% | 13.38% | 18.73% | 75.00% | 44.77% | 16.92% | 29.35% | 71.33% | 15.13% | 22.98% |
| 2015 | -5.65% | 7.13% | 3.75% | 17.54% | 185.71% | 45.38% | 0.71% | 24.40% | 32.68% | 6.77% | 11.60% |
| 2016 | 2.83% | 17.47% | 5.03% | 12.58% | 85.00% | 59.94% | -3.23% | 15.70% | 33.72% | 6.92% | 6.89% |
| 2017 | 4.56% | 6.68% | 2.62% | 13.58% | 43.24% | 60.62% | -2.14% | 12.03% | 42.54% | 5.47% | 6.90% |
| 2018 | 9.52% | 17.21% | -10.42% | 13.03% | 45.28% | 25.05% | 0.38% | 16.58% | 28.00% | -2.48% | 10.53% |
| 2019 | -20.54% | 3.03% | -12.08% | 20.38% | 67.53% | 67.07% | -8.32% | 25.94% | 23.80% | -0.47% | 13.02% |
| 2020 | -4.54% | 31.72% | 23.53% | 22.31% | 72.09% | 60.52% | -8.07% | 13.95% | 26.70% | 21.62% | 8.15% |
| **Average annual growth rate** | 2.82% | 9.22% | 8.72% | 33.05% | 47.09% | 41.92% | 12.49% | 33.44% | 48.97% | 10.57% | 17.87% |

**^*^Meta-analysis are made up of systematic review and meta-analysis., traditional meta-analysis and network meta-analysis. Sys-meta: systematic review and meta-analysis.**
